# 

**Published:** 1888-09

**Authors:** 


### The Chicago Medical Journal and Examiner.

Volume 57. Number 3.

### CONTENTS OF THIS NUMBER.

**Original Communications—**

Destruction of the Trophic Function as an Element in Determining the Localization of Infection, etc. B. Holmes ... 128

**Editorial—**

The New National Quarantine Law ... 134
The Paris Congress for the Study of Tuberculosis ... 135
Cholera and Inoculation ... 136
Yellow Fever ... 137

**Society Reports—**

CHICAGO MEDICAL SOCIETY—
Regular Meeting, May 7 ... 137
Regular Meeting, May 21 ... 139
Regular Meeting, June 4 ... 141
CHICAGO PATHOLOGICAL SOCIETY—Regular Meeting, August 13 ... 143
Alumnæ Association of the Woman's Medical College of the New York Infirmary ... 147
Annual Meeting of the British Medical Association, at Glasgow ... 150

**Extracts and Abstracts—**

ILLINOIS STATE BOARD OF HEALTH—Abstract of Report of Quarterly Meeting ... 157
Sanitary Convention at Albion, Michigan ... 162
The Formation and Excretion of Uric Acid ... 171
New Method of Treating Yellow Fever ... 176
What Kills the Children ... 178
Malignant Pustule Among Wool-Sorters ... 180
Effects of Mental Overwork ... 181
Psycological Laboratories ... 182
Lanolin and its Imitations ... 183
The Value of Diaphoresis in Infectious Diseases ... 184
Cascara Sagrada in Rheumatism ... 184
The Amalgamation of Dublin Medical Schools ... 184
Fugitive Iodism ... 185
Congenital Hernia and Hydrocele ... 185
The Value of Ampullary Dilatations, etc. ... 186
Treatment of Burns ... 186

**Book Reviews and Notices** ... 186
**Books Received** ... 188
**Pamphlets Received** ... 189
**Miscellany** ... 189
**Announcements—**

Congress of American Physicians and Surgeons ... 191

**List of Advertisers** ... x
**Publisher's Announcements** ... x and xi

